# Functional Status and Body Mass Index in Postmenopausal Women with Fibromyalgia: A Case–control Study

**DOI:** 10.3390/ijerph16224540

**Published:** 2019-11-16

**Authors:** Laura Cerón Lorente, María Carmen García Ríos, Santiago Navarro Ledesma, Rosa María Tapia Haro, Antonio Casas Barragán, María Correa-Rodríguez, María Encarnación Aguilar Ferrándiz

**Affiliations:** 1 Department of Physical Therapy, Faculty of Health Science, University of Granada (UGR), 18016 Granada, Spain; urypres@hotmail.com (L.C.L.); mcgrios@ugr.es (M.C.G.R.); snl@ugr.es (S.N.L.); rtapia@ugr.es (R.M.T.H.); antoniocb@ugr.es (A.C.B.); encaguilar@hotmail.com (M.E.A.F.); 2 Instituto de Investigación Biosanitaria ibs.GRANADA, 18014 Granada, Spain; 3 Department of Nursing, Faculty of Health Sciences, University of Granada (UGR), 18016 Granada, Spain

**Keywords:** fibromyalgia, body mass index, postmenopause, physical conditioning, disability

## Abstract

Reduced functional capacity is a common characteristic of fibromyalgia (FMS). We aimed to investigate the relationship between functional status and body mass index (BMI) in a population with and without FMS. A pilot case–control study was performed in 34 women with FMS and 22 healthy controls which were classified according to their BMI. The main outcome measures were: Balance (MiniBestest, One Leg Stance Test), functional mobility (Timed up and Go), physical disability (Health Assessment Questionnaire Disability Index), spinal range of motion (Spinal Mouse), level of physical activity at work (Leisure Time Physical Activity Instrument), and home and leisure time (Physical Activity at Home and Work). Statistical differences were observed between overweight/obese healthy controls and women with FMS for several indicators of functional capacity. FMS patients reported worse dynamic (*p* = 0.001) and static balance (right: *p* = 0.002, left: *p* = 0.001), poorer functional mobility (*p* = 0.008), and higher levels of physical disability (*p* = 0.001). Functional status is altered in FMS women compared to the healthy control group, independently of nutritional status; therefore, BMI is unlikely to play a main role in functional capacity indicators in postmenopausal FMS women. Only dynamic balance seems to reduce the obesity status in this population.

## 1. Introduction

Fibromyalgia syndrome (FMS) is a multicomponent and chronic illness whose etiology is still unknown. Current diagnostic criteria published by the American College of Rheumatology (ACR) include both pressure and widespread pain, which cannot be explained by the presence of degenerative or inflammatory disorders. Moreover, FMS includes other conditions such as cognitive behavior, restless sleep, fatigue, and somatic symptoms [1]. FMS is present in all ethnic groups [2], climates, and cultures, with the prevalence being higher in women. In the general population, the range is from 0.5% to 5%, and up to 15.7% in a clinical setting. In Spain, the estimated prevalence is 4.2% in women and 0.2% in men [3].

A more sedentary lifestyle in the FMS population results in a decrease in muscular strength and dysfunction due to the lack of activity, and affects daily, work, and leisure activities [2,3,4]. Subjects with FMS show dependence and limitations in autonomy and functionality, with postural and balance disorders being the most impactful.

Current research shows FMS to be related to a higher prevalence of being overweight or obese when compared with the general population, especially in the postmenopausal period [5]. In fact, longitudinal data from the Norwegian Nord-Trøndelag Health Study showed that being overweight or obese was associated with an increased risk of FMS, especially among women who also reported low levels of physical exercise [6]. FMS patients who are overweight or obese can worsen their symptoms [7], thus increasing their dysfunction and independence, and reducing their quality of life [4]. The available evidence supports a relationship between a higher body mass index (BMI) and a higher frequency of falls and lack of balance [8], and lower stability, strength, and flexibility [9]. These relationships, by themselves, would not explain dysfunctions caused by FMS; however, they can contribute to postural control deterioration [10,11], which affects the central system, and hence, cause higher frequencies of falls [10].

In addition, a lack of spinal movement has been related to functionality in the elderly population [12], which highlights its influence on daily tasks. Furthermore, postural control is also related to spinal mobility, and hence, activity levels and functionality [13]. The more sedentary lifestyle in the FMS population results in a decrease in muscular strength and dysfunction due to the lack of activity, and affects daily, work, and leisure activities [13].

Since reduced functional capacity is a common characteristic of FMS, and taking into account that the prevalence of overweight and obesity is high in FMS patients, especially when women reach a postmenopausal state, the aim of this study was to investigate the relationship between functional status by assessing balance, functional mobility, physical disability, hamstring flexibility, spinal range of motion, level of physical activity at work, home, and leisure time, and BMI in a population of overweight/obese and normal-weight women with and without FMS. We hypothesized that functional status is altered in FMS women compared to healthy controls, and that it depends on nutritional status.

## 2. Materials and Methods

### 2.1. Study Design and Study Population

A pilot case–control study was conducted according to the Declaration of Helsinki. The participants, a total of thirty-four women suffering from FMS and twenty-two healthy controls, provided written consent and were enrolled in this case–control study. Women with FMS were identified from the Granada Fibromyalgia Association (AGRAFIM), and controls from among the friends and relatives of the patients. An expert therapist carried out the recruitment of participants and the screening for eligibility. The inclusion criteria were as follows: aged between 34 and 64 years and FMS diagnosis according to the ACR classification criteria (modified 2010/2011) [14]. To diagnose fibromyalgia in adults, it is necessary that all the next criteria be met: (1) Present generalized pain, i.e., in at least four of five regions, (2) Present symptoms for at least 3 months at similar levels, (3) Symptom severity scale (SSS) score ≥ 5 and Widespread pain index (WPI) ≥ 7; or SSS score ≥ 9 and WPI between 4 and 6, and (4) A diagnosis of fibromyalgia does not exclude the presence of other illnesses and is valid irrespective of other diagnoses; exclusion criteria to both groups were presenting any inflammatory, neurological, or orthopedic disease which can alter balance, hearing, and vision, and cognitive impairment in terms of the ability to answer questions. Reporting was conducted in accordance with the STROBE statement [15].

### 2.2. Anthropometric Measures

Height was measured using a Harpenden stadiometer (Holtain 602VR^®^) to the nearest 0.5 cm, with participants not wearing shoes. BMI was calculated by dividing weight and height squared (kg/m^2^). Body weight and height were measured twice. The average of each measure was used for the analysis. The same trained research assistant performed all the measurements. Body mass index status was evaluated according to the World Health Organization criteria normal: 18.5 to 24.9 kg/m^2^; overweight: 25.0 to 29.9 kg/m^2^; and obese: ≥ 30 kg/m^2^). This classification was used to subdivide participants into four groups: a normal-weight group with FM, an overweight/obese group with FM, a normal-weight control group, and an overweight/obese control group.

### 2.3. Functional Status

The MiniBESTest was used to evaluate dynamic balance; it is an abbreviated version of the Bestest created by Horak et al., and therefore, has been validated [16]. The examination lasts 15 min and contains 14 individual tests. Each one is scored from 0 to 2, with 28 being the maximum result. This test has demonstrated good validity, test–retest reliability with an intraclass correlation coefficient (ICC) ranging between 0.80 and 1.26, and internal consistency with Cronbach’s alpha coefficients ranging from 0.89 to 0.96 [17]. The one leg stance test (OLST) was used to assess static balance. The test was carried out with the participant standing on one leg with their eyes open and their arms at their sides. Then, the time until the participant loses his/her balance was recorded in seconds [18]. The OLST showed a reliability for an older population of 0.89 with eyes open and 0.86 with eyes closed [19].

The Timed Up and Go Test (TUG) was used to assess general movement functionality. This test measures the time that a person takes to get up from a chair, cover a distance of 3 m, turn, and go back to the original seated position. The obtained values are classified based on the reference values for each group. Furthermore, other tasks can be added to this test to evaluate the fall risk when multitasking. Three different measurements can be obtained: the TUG, the manual TUG (the test carried out while carrying a glass of water), and the cognitive TUG (the test carried out while a cognitive activity is simultaneously carried out) [20]. The TUG test has demonstrated good inter-examiner reliability, with an ICC of 0.86, a 95% confidence interval (CI) = 0.86–0.98, and an internal consistency of 0.85 [21].

The Health Assessment Questionnaire Disability Index, (HAQ DI) which is a self-assessment questionnaire, was used to evaluate physical disability. It has 20 items grouped into 8 areas which assess the ability to carry out daily activities, and includes questions to further assess whether any help is needed to do these activities. Each item is scored from 0 to 3 (with 3 meaning maximum disability), and an overall average from all areas is obtained. This test was validated and translated into Spanish [22]. It has been reported that the reliability (ICC) of the HAQ DI in patients with FMS ranges between 0.70 and 0.77 [22] In the Spanish version, the validity and test–retest reliability were high, with a Pearson’s r > 0.4 and Spearman’s rho = 0.89 respectively [23].

A spinal mouse system was used to measure spinal range of motion. It is a non-invasive technique with which to assess the range of movement of the spine. This device is passed along the column from C7 to S3. It is carried out in different positions: standing in a neutral position, standing in a maximally-extended position, standing in a maximally-flexed position, and standing and leaning to the side. The mobility of the thoracic and lumbar areas and the total vertebral range of movement is thus measured in these positions [24]. This device showed good validity and reliability: ICC = 82 (95% IC = 0.57–0.95) [25].

The Leisure Time Physical Activity Instrument (LTPAI) was used to measure the physical activity. It has four components with three different levels of activity: light, medium, and vigorous. The obtained values indicate the number of hours in which these activity levels had been carried out each week in the last four weeks and the total number of hours of physical activity [26]. This test showed satisfactory test–retest reliability for the total score, i.e., ICC = 0.86 (CI 0.79–0.93), and for the PAHWI (ICC 0.91, CI 0.82–9.96) [26].

The Physical Activity at Home and Work (PAHWI) was used to quantify the level of physical activity at home and at work. It has three different categories: light, medium, and hard, and four categories at work: sedentary, light, medium, and hard. The obtained values indicate the number of hours spent in each activity level per week in the last four weeks, and the total number of hours of physical activity in these two areas [27]. The PAHWI instrument showed good test–retest reliability for subjects with FMS (ICC 0.91, CI 0.82–9.96) [26].

### 2.4. Statistical Analysis

SPSS® Statistics version 21.0 (IBM, Chicago, IL, USA) was used for all analyses. The Kolmogorov–Smirnov test was used to verify data distribution normality. To compare the two groups (FMS patients and heathy women) regarding sociodemographic and clinical characteristics, a Student’s t-test was performed. The Mann–Whitney U test was used to compare differences between functional status parameters and nutritional status in FMS patients and healthy women. Data were expressed as mean (Standard Deviation) for parametric tests and median (interquartile range) for non-parametric. Spearman’s correlation coefficient (r) was used to test the correlation between functional status and BMI in FMS patients and controls. *P*-values < 0.05 were considered to be statistically significant.

### 2.5. Sample Size Calculation

The sample size was calculated using the G*Power software, version 3.1.7 (University Kiel, Kiel, Germany). Based on previous published data [28], we used a Cohen’s standardized mean difference effect size of 1.20 between the FMS and healthy control groups for the Baecke Physical Activity Questionnaire, a reliable measure for functional status. According to this program, a study sample of 16 patients per group can detect a high effect size (d = 1.20) with a power of 80% at a = 0.05.

### 2.6. Ethical Approval

The local ethics committee of the University of Granada approved the study, which was conducted in accordance with the Declaration of Helsinki. Informed consent was obtained from all individual participants. Participants’ information were password protected and stored.

## 3. Results

### 3.1. Characteristics of the Study Participants

A total of 34 women with FMS and 22 healthy controls were included in this study. Table 1 shows the sociodemographic and clinical characteristics of the study population. Regarding age, weight, height, and BMI, there were not significant differences between the FMS patients and healthy women. Note that all women were postmenopausal and had a mean of 12 ± 2, 3 months of amenorrhea. As expected, the FIQ-R score was significantly higher in FMS patients than in the controls (*p* = 0.001).

### 3.2. Functional Status and Nutritional Status

Table 2 presents the median (interquartile range) and between-group differences for functional statuses in overweight/obese women and normal-weight women among the FMS patients and healthy women. Statistical differences were observed between overweight/obese healthy controls and women with FMS for several indicators of functional capacity. FMS patients reported worse dynamic (*p* = 0.001) and static balance (right: *p* = 0.002, left: *p* = 0.001), poorer functional mobility (*p* = 0.008), and higher levels of physical disability (*p* = 0.001). Overweight women with FMS also spent fewer hours in physical activity at home and work compared to overweight healthy women.

In addition, a Mann–Whitney U-test revealed significant differences between normal-weight controls and women with FMS and non-overweight for static balance (right: *p* = 0.005), functional mobility (*p* = 0.041), physical disability (*p* = 0.007), and physical activity at work and home (*p* = 0.003).

### 3.3. Correlations between Functional Status and BMI

A Spearman correlation analysis between functional status and BMI in normal-weight and overweight/obese FMS patients and healthy women is shown in Table 3; Table 4, respectively. Note that only spinal extension was positively correlated with BMI in overweight/obese healthy women (*r* = 0.749; *p* = 0.008). For the other functional capacity indicators, no significant correlations were identified.

**Figure 1:**
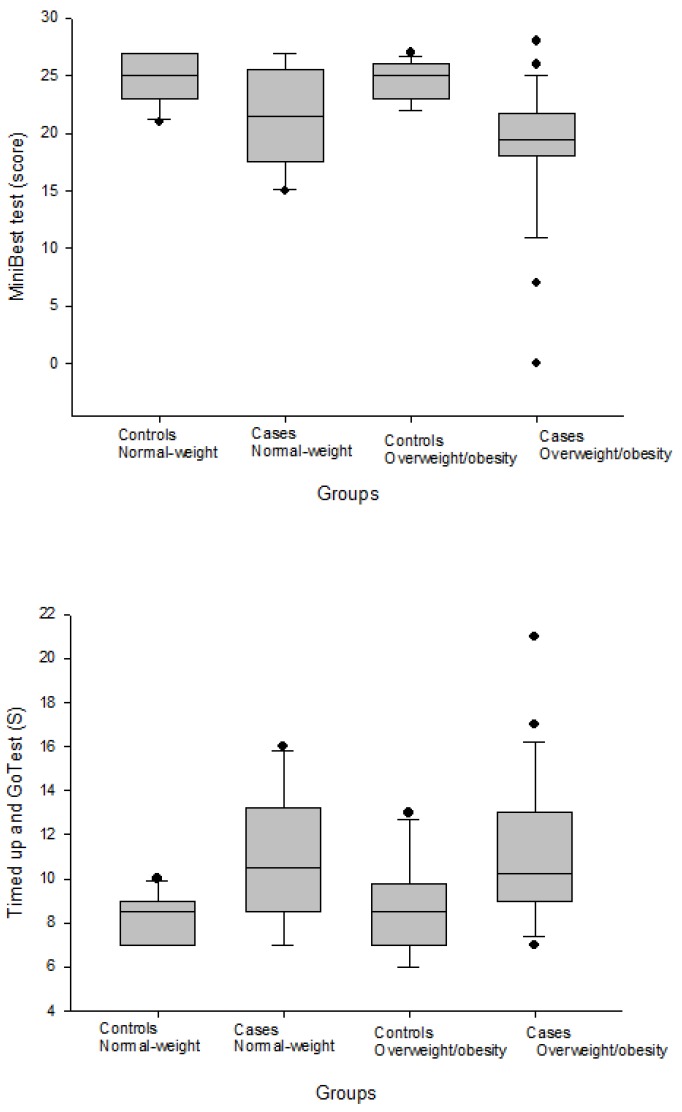
Levels of dynamic balance measured with the MiniBest and Timed up and go tests in women with fibromyalgia and healthy controls grouped by their body mass index (normal-weight versus overweight/obese). In the box plots, the boundary of the box closest to zero indicates the 25th percentile, the black line within the box marks the median, and the boundary of the box farthest from zero indicates the 75th percentile. Whiskers above and below the box indicate the 10th and 90th percentiles.

**Figure 2:**
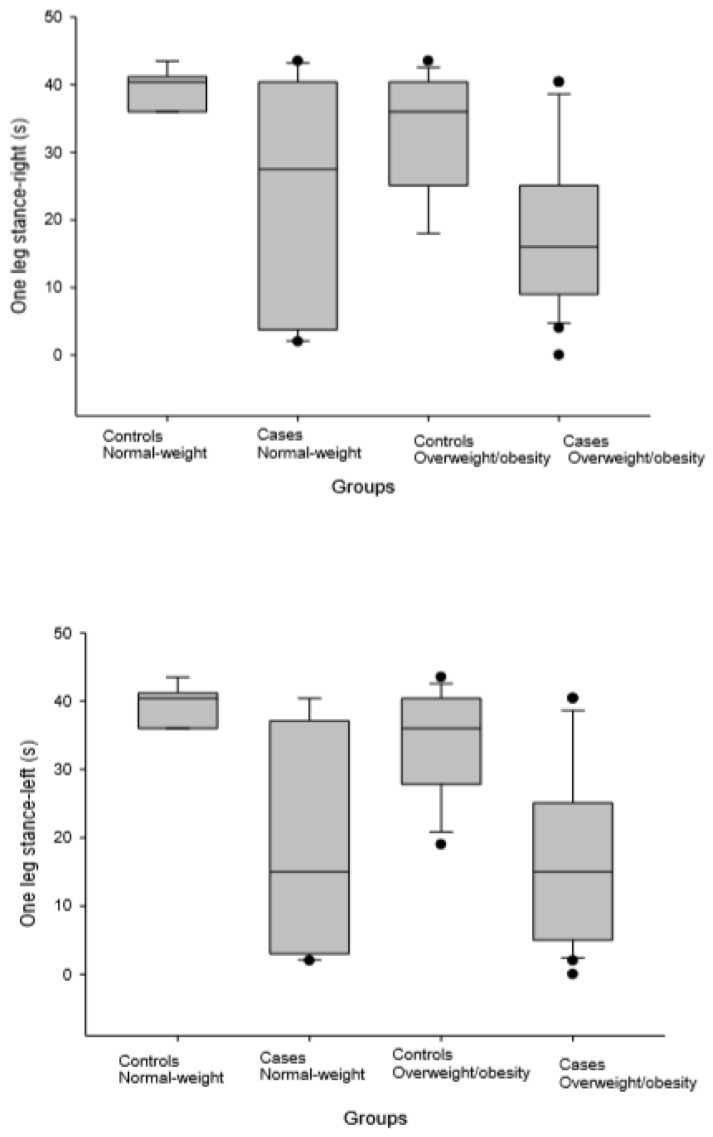
Levels of static balance measured with One leg stance test in cases of women with fibromyalgia and healthy controls grouped by their body mass index (normal-weight versus overweight/obese). In the box plots, the boundary of the box closest to zero indicates the 25th percentile, the black line within the box marks the median, and the boundary of the box farthest from zero indicates the 75th percentile. Whiskers above and below the box indicate the 10th and 90th percentiles.

Figure 1 and Figure 2 shows the levels of dynamic balance measured with the MiniBest and Timed up and go tests, and the level of static balance measured with a one-leg stance test in cases of women with fibromyalgia and healthy controls, as grouped by their body mass index (normal-weight versus overweight/obese), respectively.

## 4. Discussion

The aim of this study was to investigate the potential relationship between functional status by assessing balance, functional mobility, physical disability, hamstring flexibility, spinal range of motion, level of physical activity at work, home and leisure time, and nutritional status in a population of women with and without FMS. We found that postmenopausal, overweight/obese FMS women state showed worse dynamic and static balance, poorer functional mobility, higher levels of physical disability, and reported fewer hours of physical activity at home and work compared to overweight/obese women without FMS. In addition, our results evidenced that FMS women of normal weight also had poorer scores in static balance, functional mobility, physical disability, and physical activity at work and home that healthy women with normal weight. In agreement with a previous study, our findings suggest that several parameters of functional capacity are altered in FMS women compared to the healthy control, independent of nutritional status [29]. However, the fact that we found a difference in dynamic balance among women with and without FMS only in the overweight/obese group but not in the normal weight group might be evidence that simply avoiding obesity status may be useful advice for improving dynamic balance, and therefore, reducing fall risk in FMS women.

Despite the impaired functional status which is a characteristic of FMS, the potential association between functional capacity and obesity has barely been investigated [29,30]. A previous study concluded that there were no significant differences in most functional capacity outcomes among the obesity categories in women with FMS, suggesting that only by keeping a normal-weight status could the benefits be achieved [29]. They only showed that upper-body muscular strength and cardiorespiratory fitness, two physical indicators of functional capacity, were worse across the obesity categories in FMS women. In contrast, Carbonell-Baeza et al. concluded that weight status might play a role in the association between pain and functional-capacity levels in FMS women [30].

Nevertheless, the fact that in these studies, functional capacity was assessed by a functional fitness test battery, which differs from the battery used in the present study, makes it difficult to compare the findings. Also, it should be noted that in both studies, the lack of a group of healthy individuals further limits direct comparisons.

To our knowledge this study is the first to show differences in the dynamic balance, a relevant parameter in the evaluation of functional state, among overweight/obese FMS and healthy women. Based on this result, it may be hypothesized that a status of overweight/obesity in FMS women may lead to an increased loss of dynamic balance and muscle weakness that can cause falls. In this line, previous research conducted in the general population supports the relationship between dynamic balance and BMI [31,32,33,34]. Cancela-Carral found a significant correlation between dynamic balance and BMI in a population of older adults [31], and a recent study also reported that young obese subjects have worse balance compared to normal weight subjects [32]. Similarly, Melzer et al. indicated that obese older adults have altered characteristics of balance control, supporting the hypothesis that obesity may lead to an increased risk of instability and fall events [33]. Considering that we reported, for the first time, a significant difference in dynamic balance among overweight/obese FMS and healthy women, these results should be considered preliminary, and further studies including larger FMS populations are needed.

Our findings support the hypothesis that the functional status was impaired in obese and normal weight FMS patients compared to healthy women. In this line, the available evidence has shown that behavioral weight loss intervention might result in improvements in the quality of life and in FMS symptoms [35,36]. Regarding functional capacity, Carbonell-Baeza showed that multidisciplinary interventions alone might improve body flexibility in FMS patients [30]. Interestingly, previous case–control studies have been conducted, relating functional status and BMI to other psychological variables [37,38,39,40,41]. Sampere-Rubio et al. reported that women with FM show a significantly lower QoL than their healthy counterparts, and the factors that predict their perceived QoL are functional capacity, muscular strength, postural maintenance, pain threshold, and anxiety [37]. In this line, higher depression ranges were shown in women who suffered from FMS with respect to healthy controls, regardless of age distribution [39], and adolescents with FMS were found be more sensitive to pressure pain than their healthy peers [40]. Therefore, considering the limited data, future longitudinal studies investigating the effect on weight loss intervention regarding functional status in FMS patients are required.

This study has some potential limitations that should be acknowledged. First, due to its cross-sectional nature, casual relationships were not established. Second, since this study was conducted in a small cohort, we cannot ignore the fact that the sample size was not statistically strong enough to detect associations. Additionally, taking into account the limited study populations, stratification analysis across obesity class categories were not performed. Also, we would like to clarify that we were able to consider only normal weight and overweight/obesity as categories of BMI, since no subjects were underweight. Furthermore, our study sample consisted of a well-characterized population of FMS women and, therefore, our data might not be generalizable to other populations. Despite its limitations, the present study has its strengths. To our knowledge, this is the first study to examine the association between functional status by assessing balance, functional mobility, physical disability, hamstring flexibility, spinal range of motion, level of physical activity at work, home and leisure time, and nutritional status in a population of women with and without FMS. Also, it should be noted that a normal-weight group was included to allow direct comparisons to be made.

## 5. Conclusions

In conclusion, functional status is altered in FMS women compared to healthy control women, independently of nutritional status; therefore, BMI is unlikely to play a major role in functional capacity indicators in postmenopausal FMS patients. Only dynamic balance seems to be able to reduce the obesity status in this population. Further studies in larger study populations of FMS patients are required to validate our preliminary findings.

## Figures and Tables

**Table 1:** Sociodemographic and clinical characteristics of the study population.

Variables	Cases (*n* = 34)	Controls (*n* = 22)	*p*-Value
	Mean (SD)	Mean (SD)	
Age (years)	52.89 (7.86)	50.18 (7.50)	0.291
Weight (kg)	73.19 (14.64)	69.22 (11.94)	0.293
Height (m)	1.63 (0.09)	1.65 (0.06)	0.203
BMI (kg/m^2^)	27.69 (5.04)	25.32(3.93)	0.067
FIQ-R	63.86 (18.79)	2.64 (6.05)	0.001

BMI, body mass index; FIQ-R, Revised Fibromyalgia Impact Questionnaire. Variables are shown as mean (Standard Deviation) and a Student’s *t*-test was performed.

**Table 2:** Median (IR) and between-group differences for functional status in overweight/obese women and normal-weight women in FMS patients and healthy controls.

Clinical and Functional Status Variables	Overweight/Obese Women			Normal-Weight Women		
	Cases (*n* = 24)	Controls (*n* = 12)	*p*-Value	Cases (*n* = 10)	Controls (*n* = 10)	*p*-Value
	Median (IR)	Median (IR)		Median (IR)	Median (IR)	
Age (yeras)	54.00 (9.75)	56.00 (12.25)	0.697	47.00 (10.50)	47.50 (12.25)	0.806
Weight (kg)	76.00 (17.50)	77.00 (17.00)	0.801	58.50 (11.00)	60.00 (5.50)	0.676
Height (cm)	1.60 (0.09)	1.65 (0.14)	0.275	1.64 (0.08)	1.67 (0.07)	0.224
BMI (kg/m^2^)	28.63 (4.29)	28.61 (3.35)	0.450	21.48 (3.65)	22.05 (2.49)	0.762
MiniBESTest (score)	19.50 (3.75)	25.00 (3.00)	0.001 *	21.50 (8.00)	25.00 (4.00)	0.085
TUG (s)	10.24 (4.00)	8.50 (2.75)	0.008 *	10.50 (4.75)	8.50 (2.00)	0.041 *
TUG-manual (s)	12.00 (4.00)	10.00 (3.75)	0.037 *	12.00 (6.25)	9.00 (2.00)	0.073
TUG-cognitive (s)	13.00 (5.00)	13.00 (4.50)	0.587	15.50 (9.75)	12.50 (2.50)	0.287
One leg stance-right (s)	16.00 (16.1)	36.00 (15.3)	0.002 *	27.50 (36.65)	40.40 (5.175)	0.102
One leg stance-left (s)	15.00 (20.10)	36.00 (12.57)	0.001 *	15.00 (34.10)	40.40 (5.175)	0.005 *
HAQ-DI (score)	1.25 (0.87)	0.00 (0.75)	0.001 *	1.50 (1.12)	0.00 (0.25)	0.007 *
Spinal Flexion (°)	91.50 (30.50)	90.00 (34.00)	0.763	93.50 (24.50)	81.50 (39.25)	0.350
Spinal Extension (°)	−15.50 (−10.25)	−23.00 (−11.00)	0.087	−17.00 (13.25)	−21.50 (−5.75)	0.165
Total tange of spinal inclination (°)	109.00 (32.25)	113.00 (45.00)	0.657	114.00 (38.25)	100.50 (42.25)	0.625
LTPAI total (score)	5.00 (7.75)	5.50 (6.12)	0.755	4.50 (3.75)	6.50 (5.12)	0.081
PAHWI (total)	25.00 (32.00)	45.00 (24.75)	0.024 *	25.50 (24.25)	53.50 (30.37)	0.003 *

IR; interquartile range, BMI, body mass index; TUG: Timed up and Go; HAQ-DI: Health Assessment Questionnaire Disability Index; LTPAI: Leisure Time Physical Activity; PAHWI: Physical Activity at Home and Work. Variables are shown as median (interquartile range); a Mann–Whitney *U* test was used.

**Table 3:** Spearman correlation coefficients (r) between functional status and BMI in overweight/obese FMS patients and healthy women.

Functional Status Variables	Overweight/Obese Women with FMS (*n* = 24)		Overweight/Obese Healthy Women (*n* = 12)	
	*r*	*p*-Value	*r*	*p*-Value
MiniBESTest (score)	−0.281	0.183	−0.374	0.231
TUG (s)	−0.029	0.897	−0.322	0.307
TUG-manual (s)	−0.051	0.818	−0.201	0.531
TUG-cognitive (s)	0.162	0.460	−0.149	0.645
One leg stance-right (s)	−0.169	0.441	−0.108	0.738
One leg stance-left (s)	−0.267	0.218	0.081	0.804
HAQ-DI (score)	0.187	0.442	−0.124	0.700
Spinal Flexion (°)	−0.241	0.256	0.236	0.484
Spinal Extension (°)	−0.163	0.446	0.749	0.008
Total range of spinal inclination (°)	−0.249	0.241	0.434	0.183
LTPAI total (score)	−0.008	0.976	0.337	0.283
PAHWI (total)	0.225	0.386	0.187	0.561

TUG: Timed up and Go; HAQ-DI: Health Assessment Questionnaire Disability Index; LTPAI: Leisure Time Physical Activity; PAHWI: Physical Activity at Home and Work. Spearman’s correlation coefficient (r) was calculated.

**Table 4:** Spearman correlation coefficients (r) between functional status and BMI in normal-weight FMS patients and healthy women.

Functional Status Variables	Normal-Weight Women with FMS (*n* = 10)		Normal-Weight Healthy Women (*n* = 10)	
	*r*	*p*-Value	*r*	*p*-Value
MiniBESTest (score)	−0.269	0.452	0.181	0.617
TUG (s)	0.341	0.334	0.356	0.313
TUG-manual (s)	0.348	0.325	0.299	0.401
TUG-cognitive (s)	0.450	0.192	0.488	0.153
One leg stance-right (s)	−0.411	0.238	0.311	0.381
One leg stance-left (s)	−0.256	0.475	0.311	0.381
HAQ-DI (score)	−0.025	0.949	−0.493	0.148
Spinal Flexion (°)	0.134	0.713	−0.286	0.493
Spinal Extension (°)	−0.153	0.673	−0.479	0.230
Total range of spinal inclination (°)	0.188	0.603	−0.357	0.385
LTPAI total (score)	0.252	0.548	0.337	0.283
PAHWI (total)	0.095	0.823	0.187	0.561

TUG: Timed up and Go; HAQ-DI: Health Assessment Questionnaire Disability Index; LTPAI: Leisure Time Physical Activity; PAHWI: Physical Activity at Home and Work. Spearman’s correlation coefficient (r) was calculated.
